# Correlation of surface pressure and hue of planarizable push–pull chromophores at the air/water interface

**DOI:** 10.3762/bjoc.13.109

**Published:** 2017-06-08

**Authors:** Frederik Neuhaus, Fabio Zobi, Gerald Brezesinski, Marta Dal Molin, Stefan Matile, Andreas Zumbuehl

**Affiliations:** 1 Department of Chemistry, University of Fribourg, Chemin du Musée 9, 1700 Fribourg, Switzerland; 2 National Centre of Competence in Research (NCCR) Chemical Biology, Geneva, Switzerland; 3 Max Planck Institute of Colloids and Interfaces, Science Park Potsdam-Golm, 14476 Potsdam, Germany; 4 School of Chemistry and Biochemistry, University of Geneva, Geneva, Switzerland

**Keywords:** fluorescent probes, membrane biophysics, membrane pressure, membrane probes, monolayers

## Abstract

It is currently not possible to directly measure the lateral pressure of a biomembrane. Mechanoresponsive fluorescent probes are an elegant solution to this problem but it requires first the establishment of a direct correlation between the membrane surface pressure and the induced color change of the probe. Here, we analyze planarizable dithienothiophene push–pull probes in a monolayer at the air/water interface using fluorescence microscopy, grazing-incidence angle X-ray diffraction, and infrared reflection–absorption spectroscopy. An increase of the lateral membrane pressure leads to a well-packed layer of the ‘flipper’ mechanophores and a clear change in hue above 18 mN/m. The fluorescent probes had no influence on the measured isotherm of the natural phospholipid DPPC suggesting that the flippers probe the lateral membrane pressure without physically changing it. This makes the flipper probes a truly useful addition to the membrane probe toolbox.

## Introduction

Physical triggers are a major regulator of biological processes. The lateral bilayer membrane pressure, e.g., influences the nucleation [1] and shape changes [2] of lipid domains, it gates mechanosensitive pores [3] and globally organizes cell shape and motility [4]. However, although the surface pressure is vitally important to all living organisms, it eludes direct measurement and remains difficult to grasp.

**Figure 1:**
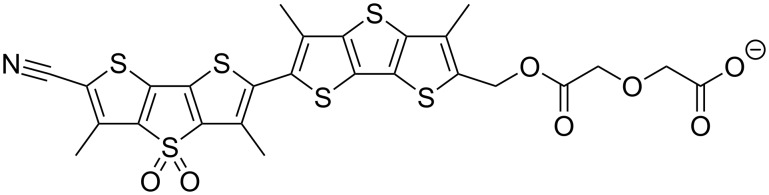
Structure of the (bis)dithienothiophene mechanosensitive flipper probe. Twisted out of planarity by two methyl groups next to the mechanosensitive bond, the two flipper-like heterocycles arrange themselves according to the surface pressure of the membrane.

The field is complicated by the fact that the lateral pressures in the inner and outer membrane leaflet do not have to be the same [5], and an indirect method of measuring the membrane pressure would only yield an averaged global value. What is needed is a probe that directly measures the local surface pressure in a single membrane leaflet. One solution to the problem are the planarizable push–pull probes that have been recently introduced. The structure of such a "flipper" probe is depicted in Figure 1 [6–9].

Without going into details, the two dithienothiophene flippers are twisted out of planarity by chalcogen bond repulsion between the methyl groups and the endocyclic sulfurs next to the mechanosensitive bond [9]. Mechanical planarization in the ground state increases the conjugation of the push–pull system. As a result, the excitation (or absorption) maximum shifts up to 80 nm to the red [8]. An anionic headgroup is added to produce an amphiphile that self-assembles into monolayers and micelles and enters directionally into lipid bilayer membranes.

In order to use fluorescent flipper mechanophores for biological measurements, it is crucial to understand the exact relation between surface pressure and their spectroscopic properties. In earlier studies [8], the fluorescence was qualitatively determined in different lipid environments: the mechanosensitive probes (1.3 mol %) were added to large unilamellar vesicles (LUV) of either DPPC (dipalmitoyl-*sn*-glycero-3-phosphocholine) or DOPC (dioleoyl-*sn*-glycero-3-phosphocholine) at different temperatures. The flipper probes in DPPC, but not in DOPC, showed a red shift of the excitation maximum while emitting the same wavelength. The important difference between these two types of vesicles is their respective membrane phase: LUVs of DPPC undergo a gel to liquid crystalline phase change at the main transition temperature *T*_m_ of 41 °C, while LUVs of DOPC remain liquid crystalline over the entire temperature range measured [10]. What is missing is a quantitative correlation between the surface pressure of a membrane and the spectroscopic properties of the flipper mechanophores [4,8]. Therefore, we have performed Langmuir–Pockels monolayer experiments.

Monolayers at the air/water interface are well known models for biological membranes, avoiding trans-bilayer leaflet correlation effects [11–14]. Various techniques exist to probe the surface pressure and the lateral organization of the monolayer [14]. Using monolayers of pure flipper probes, we were able to study the fundamental questions of surface pressure–hue correlation avoiding interfering effects from other lipids or solvents. The putative lateral organization of the hydrophobic part of the flipper probes was probed by grazing-incidence angle X-ray diffraction experiments (GIXD) [15–18], as well as infrared reflection–absorption spectroscopy (IRRAS) [19].

## Results and Discussion

### Pressure-area isotherm measurements

All pressure-area isotherm measurements were performed on Langmuir–Pockels troughs (either a self-made computer-interfaced film balance [20] using the Wilhelmy method with a roughened glass plate or the commercial film balance from Riegler & Kierstein, Potsdam, Germany, with a Wilhelmy paper plate [12] to measure the surface tension with an accuracy of ±0.1 mN/m; the accuracy of the molecular area measurements is ±0.5 Å^2^) at 295 K air and 293 K subphase temperature. Ultrapure water (18.2 MΩ·cm) has been used as subphase. A solution of the flipper mechanophore in chloroform/DMSO (8:2 vol%/vol%) was spread onto an expanded air/water interface. After evaporation and dissolution of the organic solvents, the size of the air/water interface was decreased with either one or two moving barriers (2 cm^2^/min).

**Figure 2:**
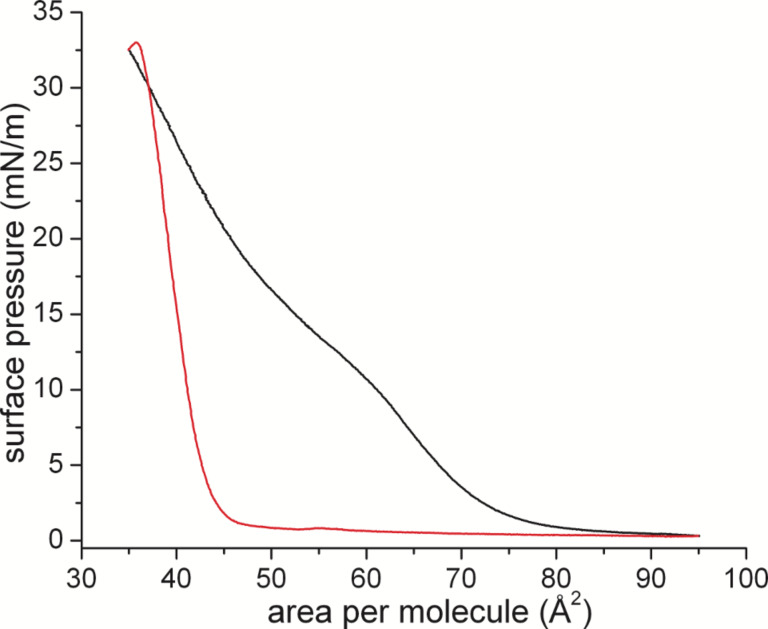
Langmuir–Pockels isotherm of a monolayer of flipper mechanophores during the first compression (black) and subsequent expansion (red) at the air/water interface at 20 °C subphase temperature and 22 °C air temperature.

The first compression curve is characterized by a fluid-like behavior (no lateral long-range ordering) at low pressure, and a transition range around 15 mN/m. Above this pressure range, the slope of the isotherm is smaller than expected for a condensed layer. However, this apparently low slope is connected with the experimental problem of measuring the surface tension of stiff films and the organization of this particular flipper mechanophore. The stiffness of the layer leads to a tilting of the Wilhelmy plate (expansion leads shortly to a surface pressure increase, seen in the red curve). The tilting of the Wilhelmy plate can be remedied by slowing down the compression speed. Under these quasi-equilibrium conditions (see Figure S1, Supporting Information File 1) it is apparent that the film is slowly being organized into a condensed phase and will remain in this same condensed phase for the remainder of the experiments.

During the first compression, the film organized obviously into a condensed phase. Upon decompression, the monolayer remains in this condensed state due to strong π–π interactions. The following compression cycles reach the exact same values as before depicting a stably organized monolayer film with possible long-range order. The area per molecule of about 38 Å^2^ is comparable to that observed for cholesterol monolayers [21–22].

### Simulation

The molecular geometry of the mechanosensitive flipper probe was simulated in the gas phase at the density functional level of theory (DFT) in order to estimate an average value of the height and area of the same (details are given in the Experimental section and in Supporting Information File 1). The calculations indicate that, in its minimum energy surface structure, the probe spans a height of 24.3 Å (see Figure S2, Supporting Information File 1). From the optimized molecular geometry, the area of the probe was calculated as 37.2 Å^2^ (see Figure S3, Supporting Information File 1). By assuming free rotation around the C–C bond connecting the two dithienothiophenes a value as high as 49.3 Å^2^ is obtained, that mirrors the area per molecule at low surface pressure found in the Langmuir–Pockels experiments. The transition into the densely packed film due to strong π–π interactions around 15 mN/m leads to the smaller area per molecule.

### Grazing incidence X-ray diffraction (GIXD)

The ordering phenomenon during the first compression can be explained by π–π interactions between the flipper mechanophores. We therefore characterized the degree of membrane ordering using synchrotron grazing incidence X-ray diffraction. The GIXD data in Supporting Information File 1 shows the absence of any long-range correlation giving rise to pronounced Bragg peaks at low surface pressures as expected from the first compression isotherm (see Figure S4, Supporting Information File 1). From the low-intensity and very broad diffraction signal, a large area per molecule of 58 Å^2^ could be calculated for the flipper probe in the monolayer between 0 and 10 mN/m. It can be concluded that the flipper mechanophores do organize in an amorphous monolayer at low pressure (akin to an ordered liquid phase). High lateral pressures could not be reached with the present set-up. Therefore, further insights were expected from monolayer IRRAS experiments.

### Infrared reflection–absorption spectroscopy (IRRAS)

**Figure 3:**
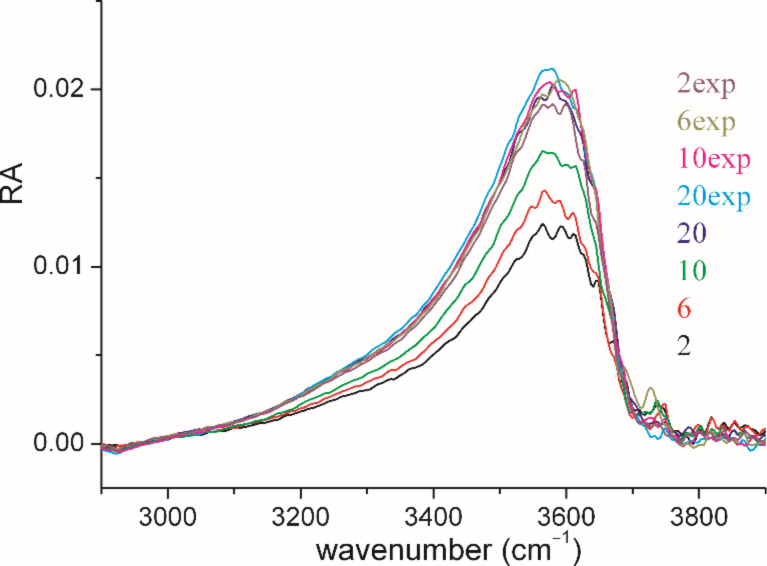
OH-stretching vibration (ν(OH); 3600 cm^−1^) for IRRA spectra of a flipper mechanophore monolayer during compression (2, 6, 10, and 20 mN/m) and expansion (20, 10, 6, 2 mN/m). The increase of intensity up to 20 mN/m indicates an increase of the effective layer thickness. It is important to note that the OH-band intensity does not change during expansion.

The infrared reflection–absorption was recorded for a monolayer at different surface pressures (see Figures S5, S6, Supporting Information File 1). The positive peak at around 3600 cm^−1^, indicating a higher intensity of the OH stretching vibrational band in the reference trough, is directly connected with the thickness of the monolayer in the sample trough (see Figure 3). The intensity of the OH-band increases during the first compression up to 20 mN/m, and remains constant at expansion. This is a clear hint that the thickness of the film increases markedly during the first compression and does not change afterwards during expansion. This experimental result can be explained by the transformation of an amorphous layer into a single layer of tightly packed molecules due to strong π–π interactions. This layer does not relax during expansion but remains tightly packed indicating the remarkable stability.

Angle dependent measurements allowed the quantification of the film thickness. The monolayer was first compressed to 20 mN/m, completely expanded and re-compressed to 10 mN/m. As shown in Figure 2, the isotherm of the expansion has the typical shape of a completely condensed film. Using a refractive index of 1.5, the value obtained from the fit of the OH stretching vibrational band (see Figure S7, Supporting Information File 1) amounts to 24.1 Å, which is in accordance with the simulated length of the molecule, suggesting that the flipper mechanophore is standing upright on the air/water interface. The strong π–π interactions stabilize this upright orientation of the flipper molecules in the monolayer. Even the expansion to 2 mN/m does not lead to changes in the condensed monolayer thickness.

It is interesting to note that the ratio of the reflection absorbance (RA) of the ν_s_(SO_2_) measured with s- and p-polarized light does not change during compression indicating no change in the orientation of this transition dipole moment (see Figure S6, Supporting Information File 1).

### Effect of flipper on DPPC

**Figure 4:**
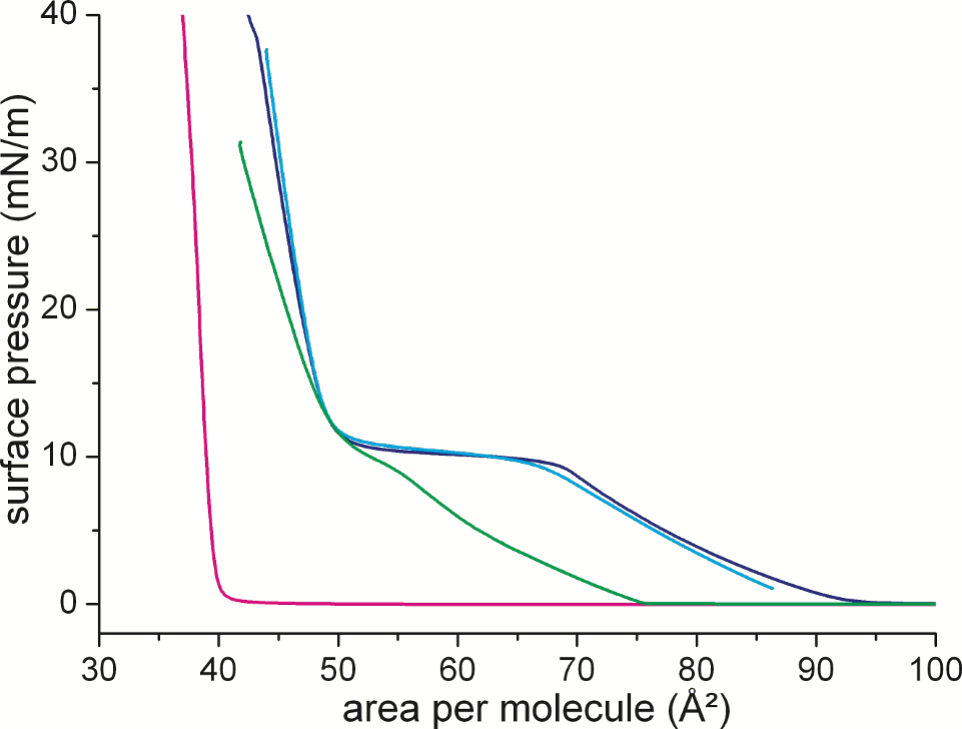
Isotherms of DPPC (dark blue), cholesterol (magenta), DPPC/cholesterol (8:2 mol/mol, green), and DPPC/flipper (8:2 mol/mol, blue) measured on water at 25 °C. The area in the mixture is given as area per DPPC molecule.

There is a structural similarity between the flipper probes and cholesterol with both molecules being amphiphilic and flat. This called for a closer look at the influence of both molecules on phospholipid membrane organization (see Figure 4).

For DPPC, the first-order phase transition between the disordered LE and the ordered LC phase can be identified as a plateau region in which the two phases are coexisting. The phase transition pressure amounts to ≈10 mN/m. Cholesterol has a fully condensed isotherm with low compressibility of the layer. The addition of flipper probes to DPPC does not influence the shape of the isotherm. This could be an indication of a lack of interactions between the two molecules. This observation is supported by the IRRAS data (ν_as_(CH_2_)) showing no influence of the 20 mol % of added mechanophore on the position of the CH_2_ stretching vibration of the DPPC chains (see Figure S8, Supporting Information File 1). This is in contrast to the influence of cholesterol. There, the isotherm is shifted and the two-phase coexistence region is hardly visible anymore. This is again in complete agreement with the IRRAS data indicating the ordering effect of cholesterol on the LE phase of DPPC (shift to lower wavenumbers) and the disordering effect (shift to higher wavenumbers) on the LC phase. Overall, the lack of influence of the flipper mechanophore on the organization of the DPPC membrane is beneficial for the flipper's purpose as a membrane probe. This paves the way for testing the correlation of the flipper's fluorescent signal and the membrane lateral surface pressure.

### Hue surface-pressure correlation

The flipper mechanophore shows a flexible geometry between the two heterocyclic chromophores. These two flippers can adapt to a decreasing monolayer molecular area and increasing surface pressure by decreasing the volume one molecule occupies. The flattening of the molecules should lead to a change of its spectroscopic properties [6–8]. In order to quantify this; we measured, to our knowledge, one of the first correlations between surface pressure and the hue of a fluorescent molecule. The hue is one of the main color appearance parameters and represents a digital value for color in the hue, saturation and value (HSV) color model.

**Figure 5:**
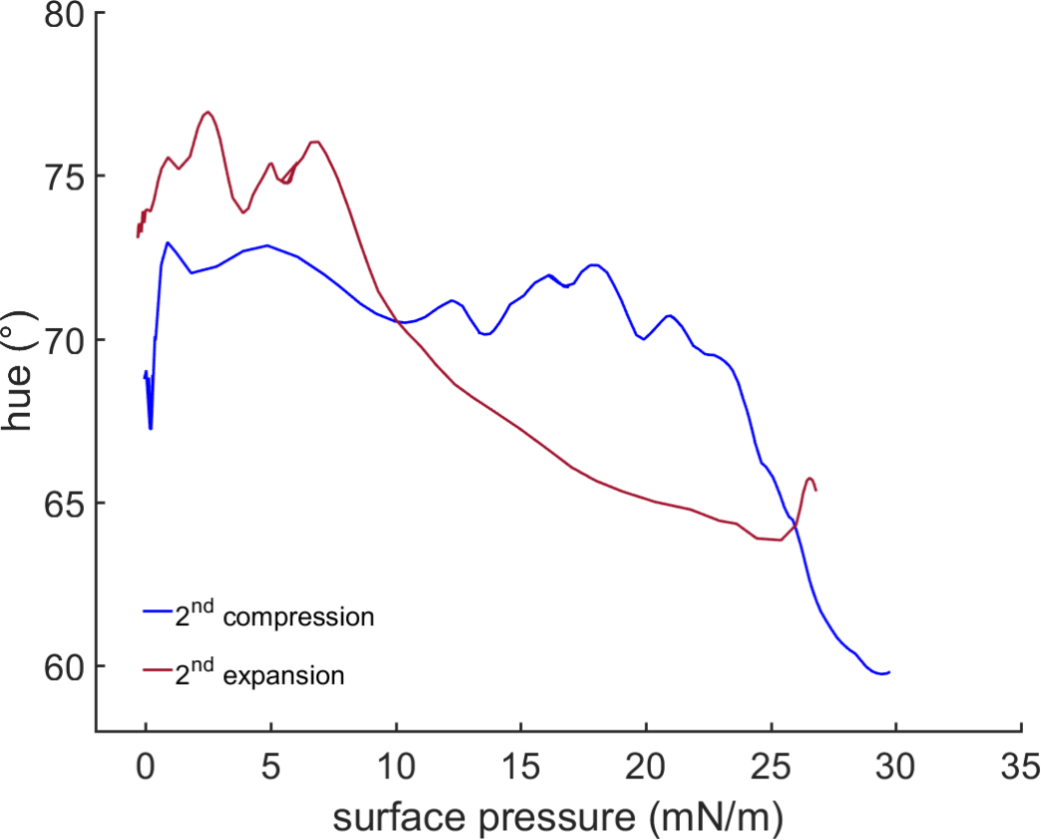
Correlation of the hue of a monolayer flipper probe with its measured surface pressure at the water/air interface at 20 °C subphase temperature and 22 °C air temperature.

In the second compression (see Figure S1, Supporting Information File 1) the correlation in Figure 5 shows a significant change in the observed hue of the monolayer starting at 18 mN/m. Although the measured areas per molecule are not fully quantitative [23], a value of 38 Å^2^ can be assumed for the flipper chromophore in the condensed state. Upon expansion, the hue relaxes back to the initial range. Compared to compression the hue relaxation on expansion is slower. This effect can be hypothesized as follows: during the compression, defects in the monolayer organization are minimized until no defects are found anymore. A small further compression then leads to an abrupt change in hue. This creates a local energy minimum. Upon expansion, again defects are introduced into the film organization with concomitant slow adaptation by the flipper mechanophores.

The color range of the change is in the orange-yellow-region of the spectrum. The lateral pressure is in the range assumed for a natural bilayer membrane (≈30 mN/m) [24–25]. The color change is significant and represents the expected red shift. Although contributions from changes in π–π stacking on spectroscopic properties cannot be excluded, the observed red shift in compressed flipper monolayers is consistent with the earlier experiments on the planarization of monomeric flipper probes in bilayer membranes of increasing order [8]. Similar interpretations have been made for the spectral changes observed upon planarization of self-assembled mechanosensitive twisted phenylethynyl polymers [26].

## Conclusion

In conclusion, we have presented the first measurement correlating the hue of a mechanoresponsive fluorescent push–pull probe to the surface pressure of its monolayer. As expected, the color changed at a surface pressure of 18 mN/m. This value ranges in the 30 mN/m that are assumed for the surface pressure of an optimally packed fluid membrane [24–25]. Compared to cholesterol, the flipper probes do not influence the membrane packing of DPPC and therefore show true potential as disturbance-free mechanosensitive membrane probes.

## Experimental

### Grazing incidence angle X-ray diffraction (GIXD)

Grazing incidence angle X-ray diffraction measurements were performed at the PETRA III/P08 beamline at the DESY-Hamburg campus, Germany. A photon beam with 15 keV was used. The monolayers were prepared on a Langmuir–Pockels trough at 295 K air and 293 K subphase temperature. Beneath the analyzed area a glass block was placed in order to dampen any mechanically induced surface movement. The trough chamber was flushed with wet helium throughout the whole measurement. The yielded data has been processed as follows; the water-data was subtracted from the flipper data to isolate the flipper signal from that of the water molecules on the surface. The weakly correlated signal was then integrated to determine the maximum position of *Q*_xy_. From the determined *d* = 2π/*Q*_xy_ the resulting area per molecule has been calculated.

### Infrared reflection–absorption spectroscopy (IRRAS)

Infrared reflection–absorption spectra were recorded on a Vertex 70 FTIR spectrometer from Bruker (Ettlingen, Germany) equipped with a liquid nitrogen cooled MCT (mercury cadmium telluride) detector attached to an external air/water reflection unit (XA-511). The IR beam was conducted out of the spectrometer and focused onto the water surface of the thermostated Langmuir trough. The measurements were carried out with p- and s-polarized light at different angles of incidence. Measurements were performed using a trough with two compartments. One compartment contained the monolayer system under investigation (sample), whereas the other was filled with the pure subphase (reference). The trough was shuttled by a computer-controlled shuttle system to illuminate either the sample or the reference [19,27–28]. The single-beam reflectance spectrum (*R*_0_) from the reference trough was taken as background for the single-beam reflectance spectrum (*R*) of the monolayer in the sample trough to calculate the reflection–absorption spectrum as −log(*R*/*R*_0_) in order to eliminate the water vapor signal. In order to maintain a constant water vapor content, the whole system was placed into a hermetically sealed box. The resolution and scanner speed in all experiments were 8 cm^−1^ and 20 kHz. The incident IR beam was polarized with a KRS-5 wire grid polarizer. For s-polarized light, spectra were co-added over 200 scans, and spectra with p-polarized light were co-added over 400 scans. Spectra were corrected to a common baseline to allow for comparison. IRRA spectra were simulated using a MATLAB program [29–30] on the basis of the optical model of Kuzmin and Michailov [31–32]. The intensity and shape of a reflection absorption band depend on the absorption coefficient *k*, the full-width of half-height (fwhh), the orientation of the transition dipole moment (TDM) within the molecule α, the molecular tilt angle θ, the polarization and the angle of incidence (AoI) of the incoming light, as well as the layer thickness *d* and its refractive index *n*. Simulated spectra were fitted to the experimental data in a global fit, where all spectra recorded at different AoI and different polarizations were fitted in one non-linear least square minimization using the Levenberg/Marquardt algorithm. The polarizer quality was set to Γ = 0.01. The optical constants of the water subphase were taken from Bertie et al. [33–34]. The layer thickness *d* was determined from a fit of the OH stretching vibrational band (ν(OH)) in the range of 3800–3000 cm^−1^.

### Computational simulations

Geometry optimization, as well as frequency calculations for the flipper mechanophore, were performed in the gas phase at the density functional level of theory with the Gaussian 03 program package [35] using the hybrid B3LYP functional [36] in conjunction with the LanL2DZ basis set [37–39]. The geometry of the flipper mechanophore was fully optimized without symmetry restrictions. The nature of the stationary points was checked by computing vibrational frequencies in order to verify true minima. The final optimized geometry shows no negative values of vibrational frequencies. The height (*h*) and the minimum area (*A*) value of the flipper mechanophore were measured on the basis of the structural parameters of the optimized geometry. These were respectively obtained by: a) measuring the distance between the oxygen and nitrogen atoms of the terminal carboxylic and ethynyl groups (*h*) and b) measuring the distance *d* between centroids of planes defined by the outmost external atoms with *A* = π(*d*/2)^2^. In the gas phase optimized geometry *d* = 6.88 Å giving *A* = 37.2 Å^2^. However, by assuming free rotation around the C–C bond connecting the two dithienothiophene units a maximum value of *d* = 7.92 Å is obtained giving *A* = 49.3 Å^2^.

### Hue measurement

The optical signal from the Langmuir–Pockels trough was recorded with a Leica DFC7000 T microscope camera. The optics was provided by a home-made fluorescence microscope (Riegler & Kierstein, Germany). The video processing was performed using a self-developed script running on MatLab^®^ R2015a (Version: 8.5.0.197613), which also correlated the data of the pressure/area isotherms. The hue values were calculated from the RGB (red-green-blue) data recorded from the microscope camera using the following equations via the rgb2hsv functionality of MatLab^®^ R2015a:


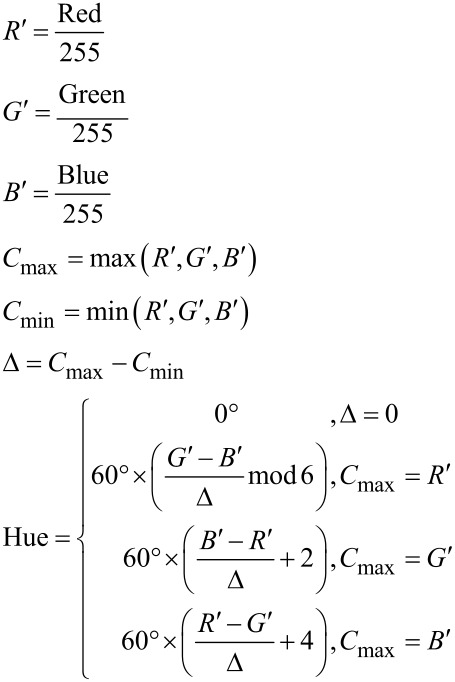


The curves were fitted using a Lowess regression which is a local regressiong using weighted linear least squares and a second degree polynomial model giving no weight to outliers higher than sixfold absolute mean.

## Supporting Information

File 1 Surface pressure/area per molecule isotherms, energy minimized structures of the flipper mechanophore, GIXD heightmaps, and IRRAS data.
