# Supplementary material for: Correlation of surface pressure and hue of planarizable push–pull chromophores at the air/water interface
Source: Beilstein J Org Chem. 2017 Jun 8;13:1099–105. doi: 10.3762/bjoc.13.109 (PMC5480347; doi:10.3762/bjoc.13.109)
Supplement: File 1 — Surface pressure/area per molecule isotherms, energy minimized structures of the flipper mechanophore, GIXD heightmaps, and IRRAS data. [file Beilstein_J_Org_Chem-13-1099-s001.pdf]

Supporting Information

for

Correlation of surface pressure and hue of planarizable push–pull chromophores at the air/water interface

Frederik Neuhaus[1,2], Fabio Zobi[1], Gerald Brezesinski [3], Marta Dal Molin[2,4], Stefan Matile[2,4] and Andreas Zumbuehl[1,2]*

Address: [1]Department of Chemistry, University of Fribourg, Chemin du Musée 9, 1700 Fribourg, Switzerland, [2]National Centre of Competence in Research (NCCR) Chemical Biology, Geneva, Switzerland, [3]Max Planck Institute of Colloids and Interfaces, Science Park Potsdam-Golm, 14476 Potsdam, Germany and [4]School of Chemistry and Biochemistry, University of Geneva, Geneva, Switzerland

Email: Andreas Zumbuehl - andreas.zumbuehl@unifr.ch

* Corresponding author

Surface pressure/area per molecule isotherms, energy minimized structures of the flipper mechanophore, GIXD heightmaps, and IRRAS data

Surface pressure/area per molecule isotherms

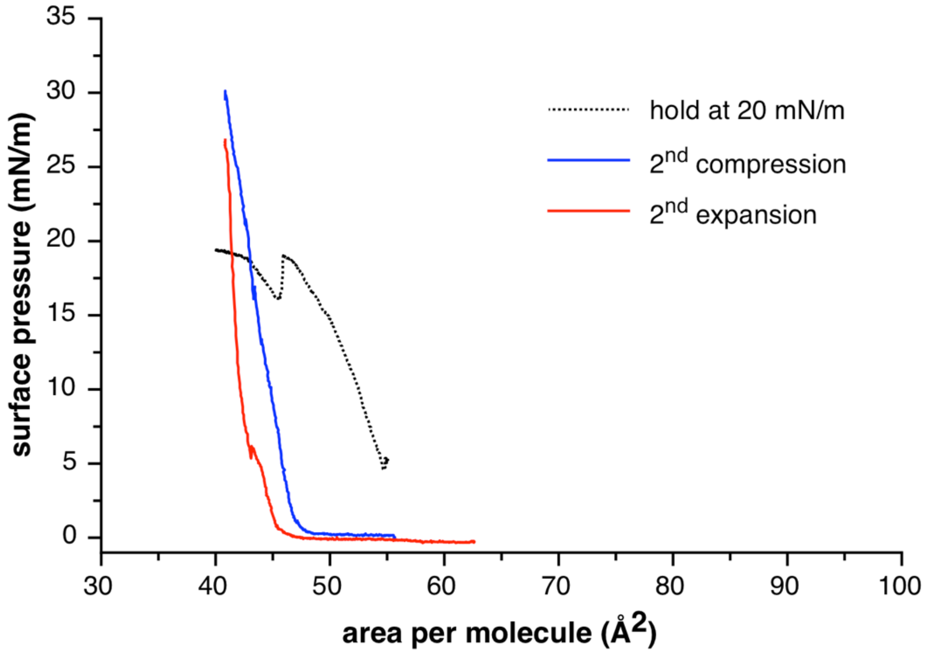

Figure S1: Composite plot of Langmuir–Pockels isotherms of the flipper mechanophore monolayer during consecutive compression and expansion at the air/water interface at 20 °C subphase temperature and 22 °C air temperature. The axes were adopted to the same size as in Figure 2. The dotted line represents the first compression measured separately under quasi-equilibrium conditions: the film balance was set to keep 20 mN/m at all time. This means that the film was compressed with decreasing velocity until a constant value of 20 mN/m was reached; this value was then kept constant. A phase change is apparent at 46 Å^2 and the curve reaches the same area per molecules found in the condensed phase of the other experiments.

The other curves are the curves measured during the recording of the color hue change. In order to reduce the amount of data for processing, the film was compressed at 7.5 cm^2/min which was faster compared to Figure 2. This leads to a small hysteresis between compression and expansion curves.

Computational simulation

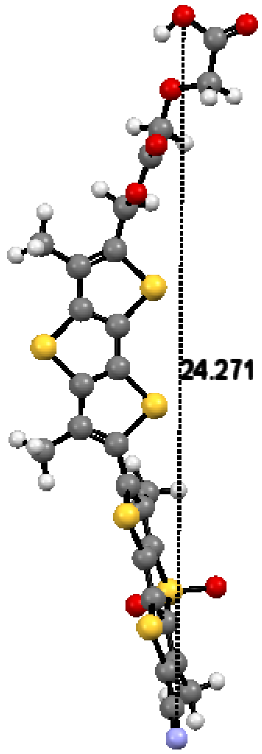

Figure S2: Simulation of the length of the flipper mechanophore. The length matches the length of the molecule calculated from monolayer experiments.

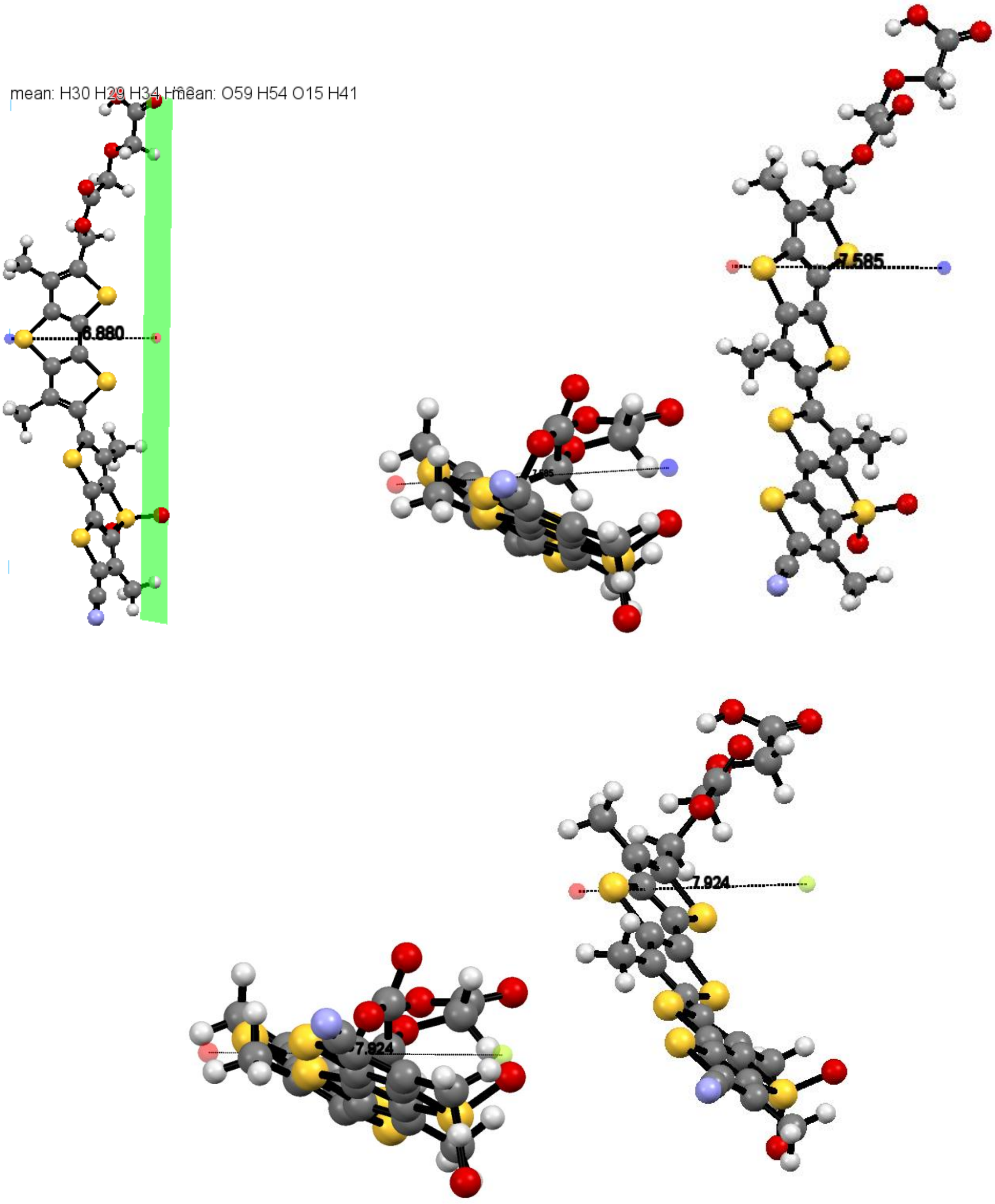

Figure S3: Possible maximal lateral molecular diameter of 7.92 Å for the flipper mechanophore from gas phase DFT calculations.

Grazing incidence X-ray diffraction (GIXD)

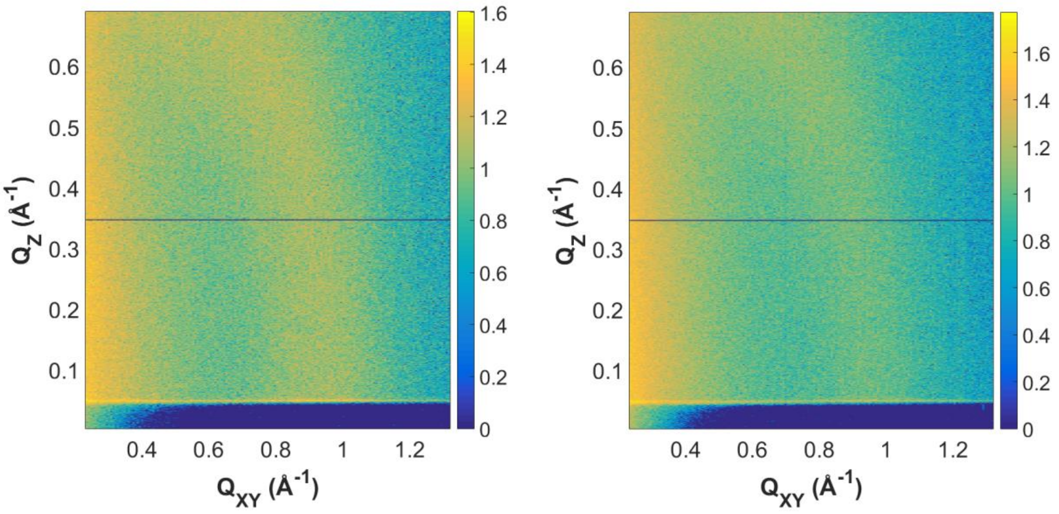

Figure S4: Scattered intensity (heightmap) as a function of the horizontal (Q_{xy}) and vertical (Q_z) components of the scattering vector of a flipper mechanophore monolayer at 3 mN/m (left) and water (right) showing arc like intensity distributions. No ordering of the flipper mechanophore molecules was found in the monolayer at low lateral pressure. Subtracting the water-heightmap from the flipper-heightmap shows a weak signal at Q_{XY} = 0.887 Å^{-1} corresponding to an area per molecule of 57.9 Å^2.

Infrared reflection-absorption spectroscopy (IRRAS)

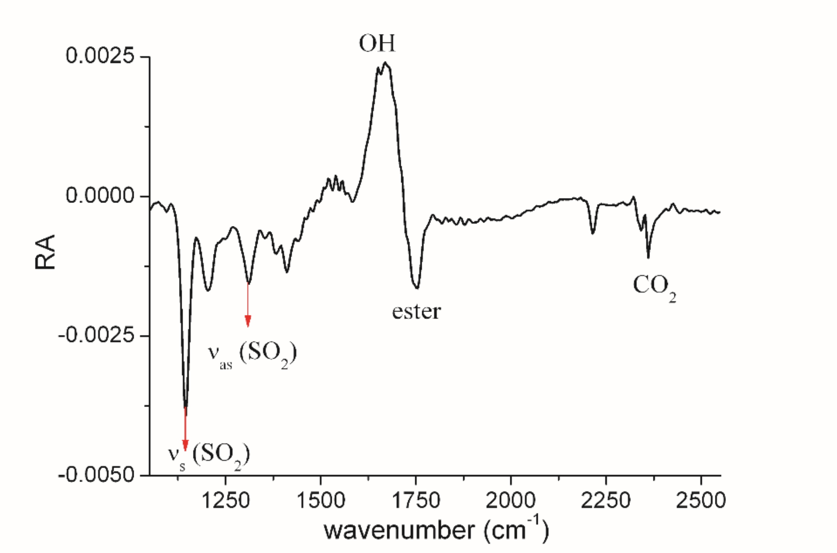

Figure S5: Selected region of the IRRA spectrum of the flipper mechanophore measured at 20 mN/m.

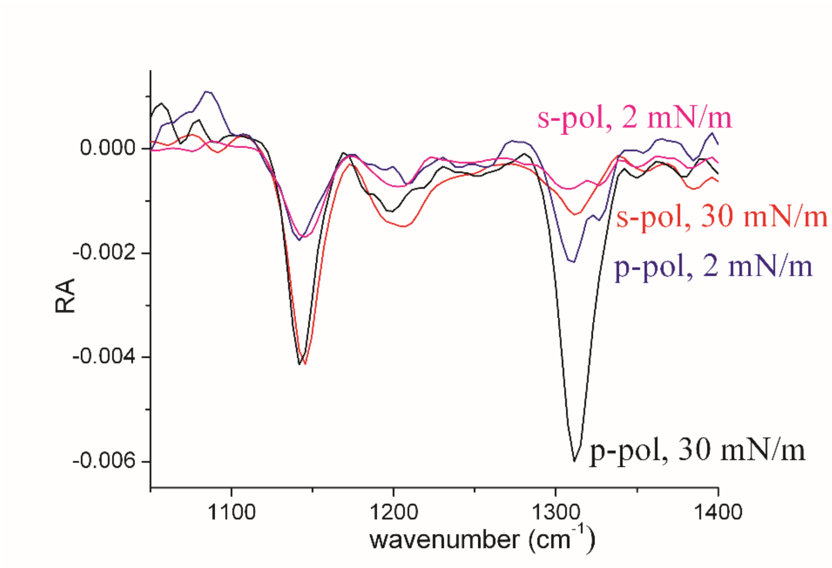

Figure S6: Selected region ($v_s(SO_2)$ and $v_{as}(SO_2)$) of the IRRA spectrum of the flipper mechanophore measured at different surface pressures with s- and p-polarized light, respectively.

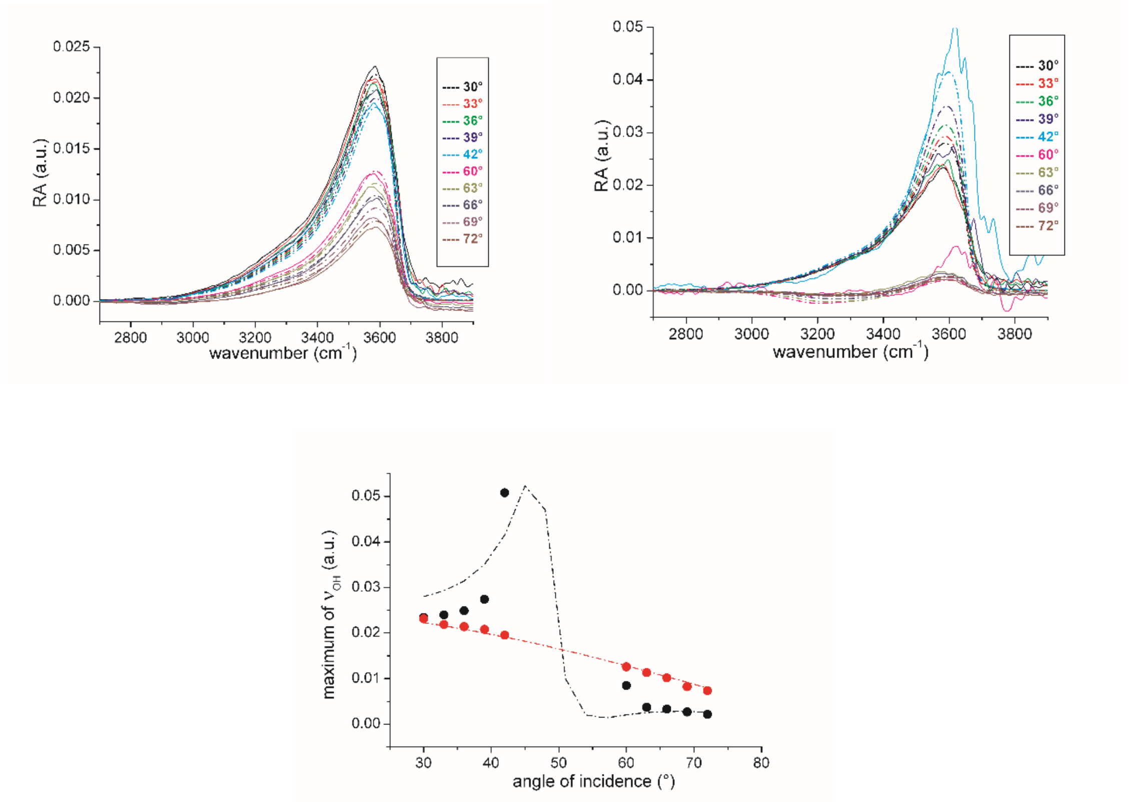

Figure S7: Simulation and fit of the OH-stretching vibration of the subphase (v_{OH}; 3600 cm^{-1}) for IRRA spectra of a monolayer at 10 mN/m (second compression curve). The best fit was achieved with a layer thickness of d = 24.1 Å and a refractive index of the layer of n = 1.5. The experimentally measured spectra (solid line) and simulated spectra (dashed-dotted line) for s-polarized light (top left) and p-polarized

light (top right) at different angles of incidence are shown. The maxima of the experimentally measured (dots) and simulated bands (line) of the OH-stretching vibration for p-polarized light (black) and s-polarized light (red) are presented as a function of the angle of incidence (bottom).

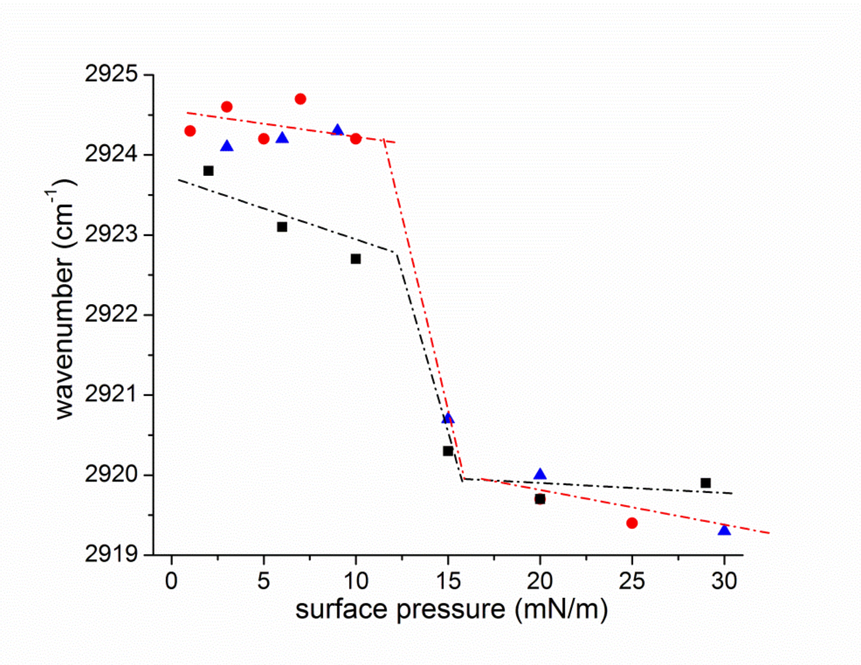

Figure S8: Wavenumbers of the asymmetric CH_2 stretching vibration vs. lateral pressure of monolayers on water at 25 °C: DPPC (▲), DPPC + 20 mol % Chol (■), DPPC + 20 mol % flipper mechanophore (●).
